# Clinical and Radiographic Assessment of Reasons for Replacement of Metal- Ceramic Fixed Dental Prostheses in Patients Referring to Dental School

**DOI:** 10.4317/jced.53850

**Published:** 2018-01-01

**Authors:** Roa’a Al Refai, Samah Saker

**Affiliations:** 1 School of Dentistry, Taibah University, KSA; 2 Associate Professor, Fixed prosthodontics Department, Faculty of Dentistry, Mansoura University, Egypt

## Abstract

**Background:**

The expected length of service and reasons for fixed dental prostheses (FDPs) replacement are a frequent inquiry by patients while the answers were mainly based on studies reports that was conducted outside the middle east region. This clinical and radiographic survey was constructed to assess and survey clinically and radiographically the reasons of replacement of metal-ceramic fixed dental prostheses, amongst patients reporting at dental school in Taibah University.

**Material and Methods:**

Between January and May 2016, 151 patients were recruited for this study. Interview (include questions pertained to the length of service of the prosthesis, the nature of complaint as told by patient in her own words), clinical examination, intra-oral photographs, and periapical radiographs, were done by the researchers. The parameters assessed were secondary caries, open margins, loss of retention, failure of endodontic treatment of the abutment and periodontal diseases.

**Results:**

A total number of 249 failed fixed dental prostheses were evaluated. Of which 180 (39.7%) were single crowns, 159 (35.0%) were retainers and 117 (25.8%) were pontics in 69 fixed partial denture. The most common reason for replacement of fixed restorations was periodontal diseases affecting 92.8% of all types’ restorations, followed by defective margin in 90.4% of examined restoration, poor aesthetic in 88% of restorations, while periapical involvement was found in 85.5% of fixed dental prosthesis. The survival rates of fixed prostheses were not predictable, and no association was found between number of years in service and the number of restorations.

**Conclusions:**

The most common reasons for replacing single unit fixed dental prostheses are periodontal diseases and periapical involvement, while defective margins and poor aesthetic mainly associated with multi-unit fixed dental prostheses.

** Key words:**Failure, Fixed dental prosthesis, Survival, Replacement.

## Introduction

Replacing missing teeth by means of fixed dental prosthesis is a very common treatment modality in dentistry. Fixed dental prosthesis provides satisfaction for the patient and the dentist due to its stability, retention and availability. Rational for replacing missing teeth by fixed prostheses is to improve patient comfort and increase mastication efficiency, maintain the health and integrity of the remaining alveolar ridge, and elevate the patient’s psychological status (1-3).

To achieve such criteria, multiple factors must be kept in mind while planning and designing fixed prosthesis, starting with proper case selection, treatment planning, and considering all biological, mechanical and esthetic factors before beginning this way of treatment. Giving attention to all aspects mentioned above will lead to better result with favourable longevity of the prosthesis. Otherwise, failure and clinical complications might be a possibility (2). A good knowledge about these complications will be of great value for clinicians to establish a treatment plan, design and choose the right material for the patient. And will be helpful for the success of the prosthesis to reach optimum satisfaction (3).

A complication has been defined as “a secondary disease or condition developing in the course of a primary disease or condition.” Even though complications could be a sign that clinical failure has occurred, but this is not always true. Complications mostly are conditions that occur either during or after an appropriate fixed prosthodontic treatment have been performed (4,5).

It is confirmed in many literature, that various clinical complications were responsible for failure of fixed dental prosthesis, although the use of specific clinical, radiographic, and technical measures may have improved the length of service for fixed prosthesis. For example, removal of pre-existing restoration on the abutment, increase the percentage of gold in the alloy, placing the margin of the restoration coronal to the gingival crest, and periapical radiograph was taken prior to cementation to insure fitting of proximal margin. However, the main cause remained the same over the past years, which is dental caries, occurring in (38%) of patients (6).

Walton *et al.*, reported that the mean length of service of all prosthesis evaluated in the study was 8 years. Dental caries was the most observed cause of failure, affecting 22% of the units failed and leading to the necessity for replacement (7).

Other causes of failures include poor aesthetics, technical problems (fractures of the fixed connector, porcelain fractures, wear of occlusal surfaces), failure of root canal treatment of the abutment teeth, and periodontal diseases (8-18).

Now it is important to screen the recent patterns of changes, and decide the particular reasons of failure which necessitate replacement, to provide dentists with profitable information for prognosis and avoiding the common prosthodontics complications.

As the metal- ceramic fixed dental prostheses still used for teeth replacement in Arabic countries as a result of socio-economic factors, it’s important to monitor and assess complications correlated with failure of metal ceramic fixed dental prostheses. This clinical and radiographic survey was constructed to assess and survey clinically and radiographically the reasons of replacement of fixed dental prostheses, amongst patients reporting at dental school in Taibah University and to assess the survival rates of FDPs in patient reported in dental clinics at Taibah University.

## Material and Methods

This is a retrospective observational descriptive cross sectional study. 151 Patients were recruited for this study with an age range between 20 to 60 years. They were examined between January and May 2016. This study was approved by the Ethics committee of Taibah University(TDU-REC). All the participants included in the study provided written informed consent before participation. The patients were Interviewed (include questions pertained to the length of service of the prosthesis, the nature of complaint as told by patient in her own words), clinically examined (using dental mirror, explorer, tweezer and periodontal probe), intra-oral photographs (using Carestream intra-oral camera or Canon D450 SLR) and periapical radiographs (using Carestream digital radiograph system), were taken. The parameters assessed were open margins, loss of retention, periodontal diseases, secondary caries and failure of endodontic treatment of the abutment.

FDPs failure classifications were based on those reported by Walton *et al.* (19) ( Table 1).

**Table 1:**
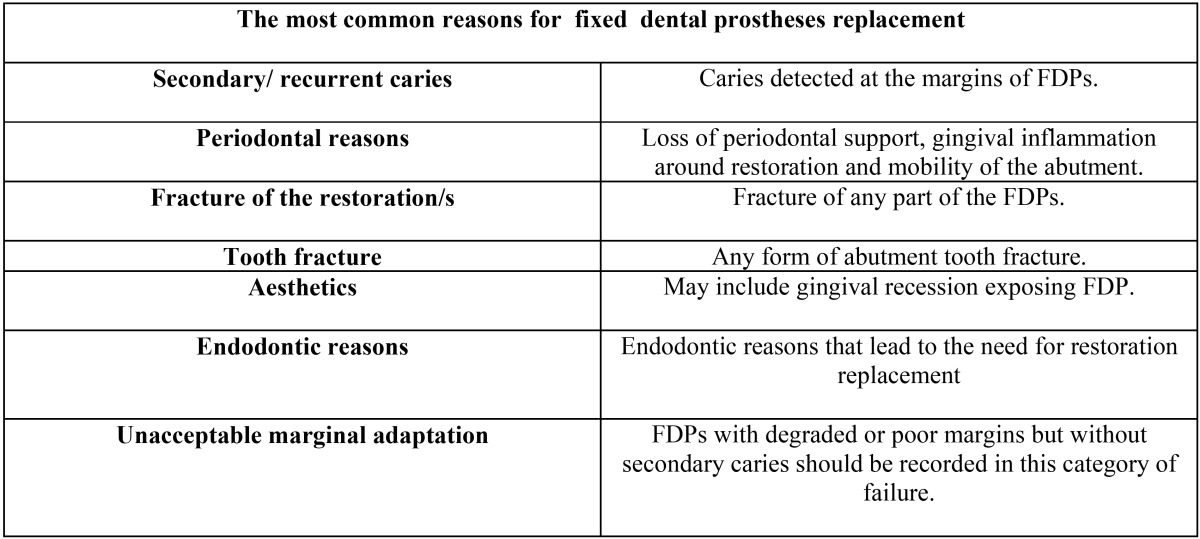
Criteria of the failed fixed dental prostheses.

-Data collection and analysis

Data were collected during clinical examination by researcher and entered using R4 system (which is used in the clinics), then coded and entered into Microsoft excel software. Data was analysed by Statistical Package for the Social Sciences (SPSS). The chi (x2) criterion was used to evaluate qualitative data. The level of significance of 0.05 is chosen to assess the statistical hypotheses.

## Results

A total number of 151 patients with failed fixed dental prosthesis were examined in two months. Total number of failed fixed dental prosthesis was 249, containing 453 units. Of which 180 (39.7%) were single crowns, 156 (34.4%) were retainers and 117 (25.8%) were pontics in 69 fixed partial denture. The most usual abutments were canines in maxillary arch and molars in mandibular arch.

**Figure 1:**
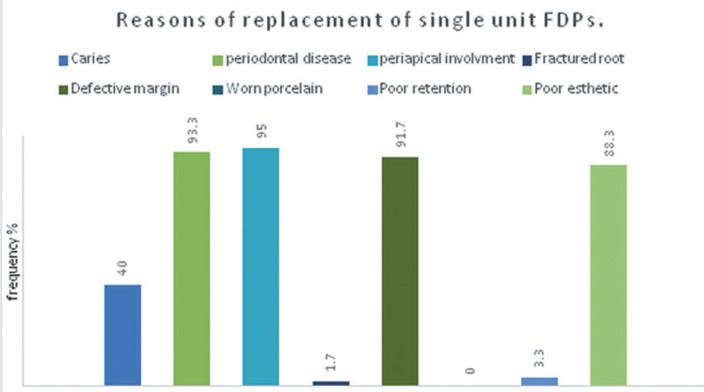
Reasons of replacement of single- unit FDPs.

**Figure 2:**
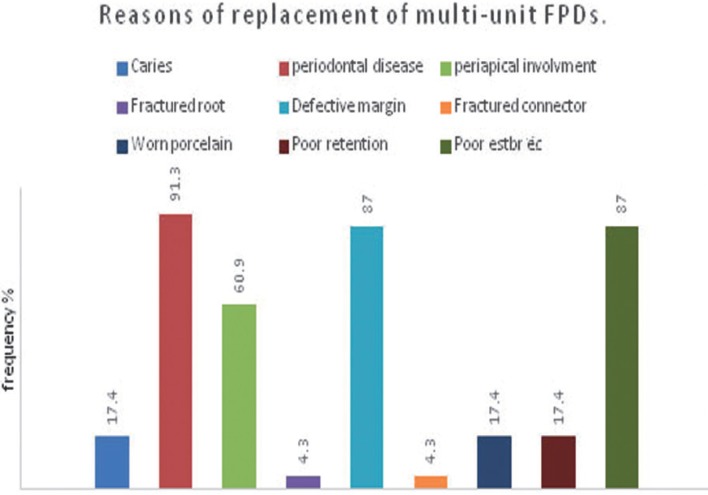
Reasons of replacement of multi-unit FDPs.

**Figure 3:**
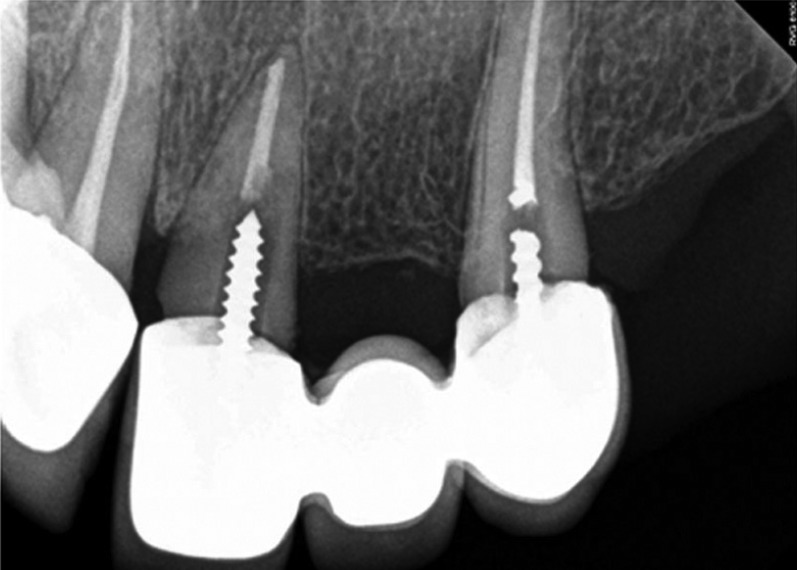
Radiographic picture of a failed FDP as a result of failed posts.

The most common reason for replacement of multi-unit fixed dental prostheses were periodontal diseases affecting 92.8% of restorations, followed by defective margin in 90.4% of examined restoration, poor esthetic in 88% of restorations, while periapical involvement was found in 85.5% of examined fixed dental prosthesis. While caries and periapical involvement were mainly associated with crowns and the difference was statistically significant (*p*=0.000). On the other hand, worn porcelain and loss of retention were mainly associated with fixed partial denture, the difference was statistically significant (*p*=0,001), ( Table 2, Figs 1-3.)

**Table 2:**
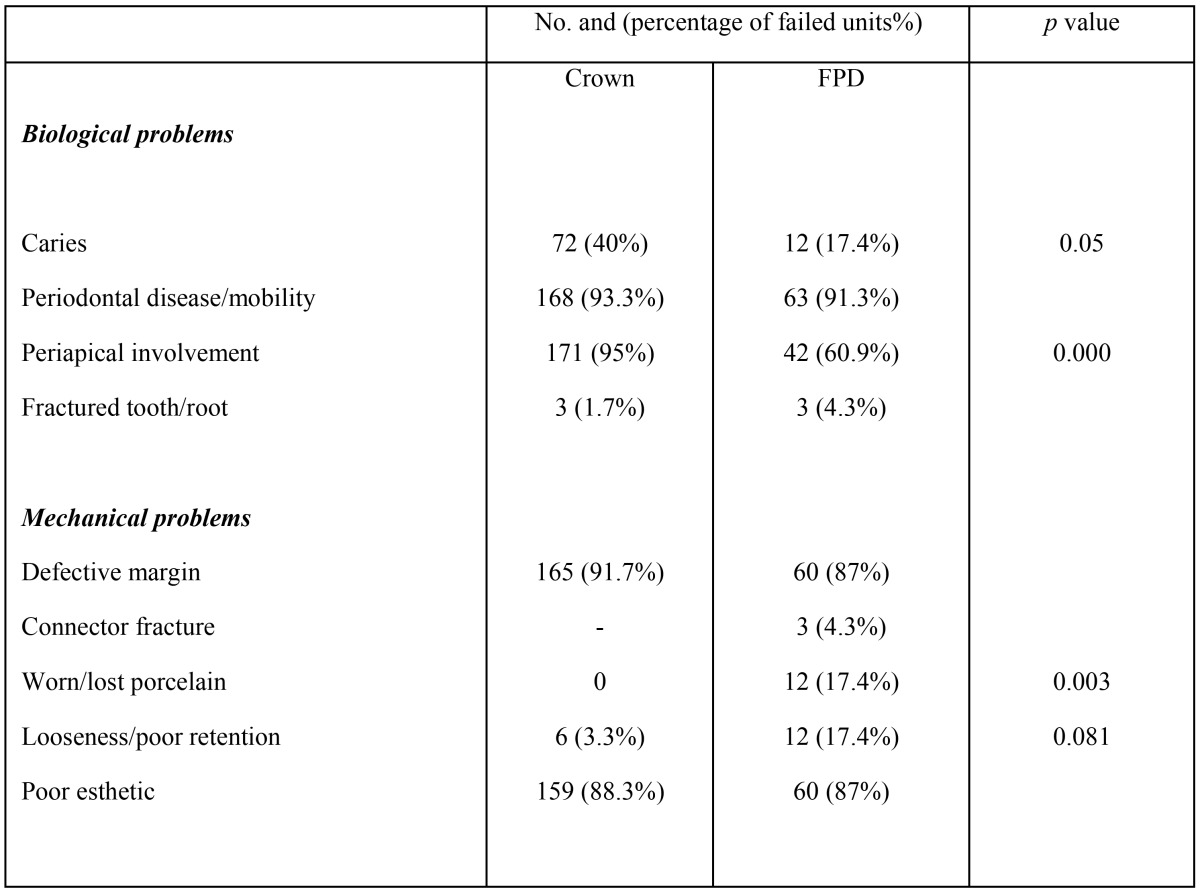
Reasons for replacement of fixed dental prostheses.

The Mean length of service of single and multi unit fixed dental prostheses were presented in  Table 3, Table 4.

**Table 3:**
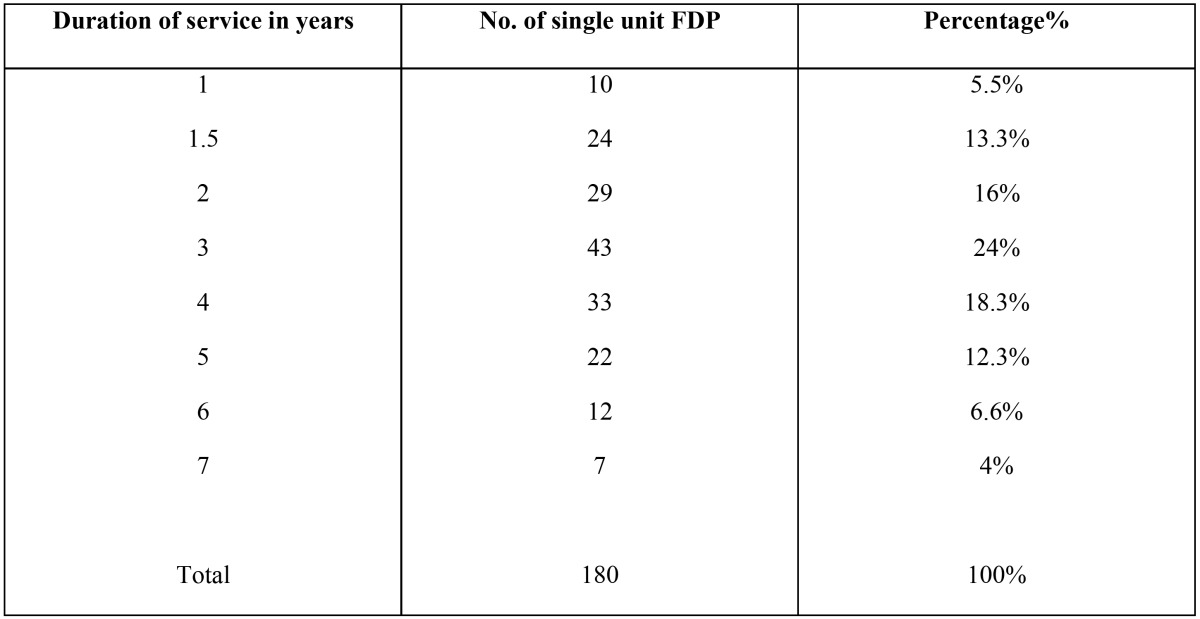
Mean Length of service of fixed crown.

**Table 4:**
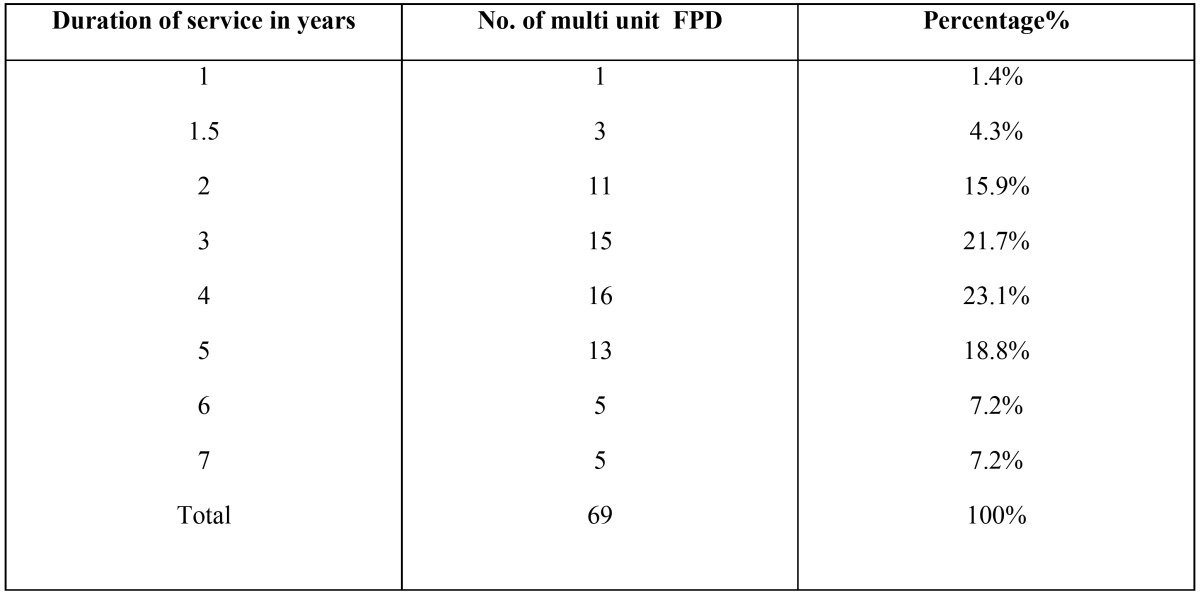
Mean Length of service of FPDs.

There is no correlation between years in service and the survival of restoration; it mainly depends on the marginal fitting of the restoration, quality of root canal treatment under restoration, maintenance and recall visits.

## Discussion

This study was conducted to investigate the reasons of failure of metal-ceramic fixed dental prostheses, and to assess the survival rates of FDPs in patient reported in dental clinics at Taibah University.

The result of this study revealed that, the major cause of single unit FDPs failure resulted from dental caries and periapical involvement, this findings was supported by the findings of Goodacre *et al.* (3) and Walton *et al.*, (7,19,20) who reported that, the most common cause of failure of single unit fixed dental prostheses is due to caries, while porcelain fracture and loss are more significant in patient with multi unit FDPs.

Several risk factors may have influenced the occurrence of dental caries in association with fixed dental prostheses, comprise existing dental caries, heavily restored dentition, the size of marginal gap, home hygiene, and frequency of professional prophylaxis (20-22).

Fixed dental prostheses may affect the conditions of periodontal tissues, the incidance of caries and the amount of stress on abutment teeth (20-24).

Radiographs may be helpful in evaluating interproximal margins between abutments,

as the clinical evaluation is often difficult. Although radiographs are two-dimensional images, they may provide enhanced analysis of interproximal marginal adaptation when combined with clinical evaluation.

Regarding the survival rates and longevity of the examined FDPs, there was no correlation between the number of year in service and survivability of FDPs. These results are similar to the finding of Libby *et al.*, as they concluded that the number of years in service provided no information on predictability of failure for FDPs (6).

Possible limitations of the study might be that the place of initial construction was not included during obtaining data, which could be significant as different dentists and technicians with varying skills have operated on the patients. Another issue is single study site, as it was difficult for the researcher to collect information, examine and evaluate patients in other centres.

Recommendation: Investigations of the initial reasons of placement of fixed dental prosthesis is recommended, as there were some cases of no reasonable cause of placement of restoration in the first place. Future studies should take the place of construction of the prosthesis in consideration (governmental services, private centres, Universities clinics).

## Conclusions

The most common reasons for replacing single-unit FDPs are periodontal diseases and periapical involvement, while defective margins and poor esthetic mainly associated with multi-unit FDPs.
